# Cytoskeletal tropomyosins: choreographers of actin filament functional diversity

**DOI:** 10.1007/s10974-013-9355-8

**Published:** 2013-08-01

**Authors:** Howard Vindin, Peter Gunning

**Affiliations:** Oncology Research Unit, School of Medical Sciences, University of New South Wales, Sydney, NSW Australia

**Keywords:** Tropomyosin, Cytoskeleton, Actin, Cytoskeletal regulation

## Abstract

The actin cytoskeleton plays a central role in many essential cellular processes. Its involvement requires actin filaments to form multiple populations with different structural and therefore functional properties in specific subcellular locations. This diversity is facilitated through the interaction between actin and a number of actin binding proteins. One family of proteins, the tropomyosins, are absolutely essential in regulating actin’s ability to form such diverse structures. In this review we integrate studies from different organisms and cell types in an attempt to provide a unifying view of tropomyosin dependent regulation of the actin cytoskeleton.

## Introduction

The actin cytoskeleton is a diverse system involved in a plethora of cellular functions including adhesion, cytokinesis, cell motility, contractile force, signaling, intracellular transport and apoptosis. There is now a mounting body of evidence demonstrating that the ability of one filament system to perform such a remarkable range of functions is facilitated through the functional specification of actin filaments by their associated tropomyosin isoform(s) (Gunning et al. 2005). Historically tropomyosin has been referred to as muscle or non-muscle. However, muscle has been shown to express tropomyosin localized to the actin cytoskeleton that is distinct from those present in the contractile apparatus (Kee et al. 2009). Therefore, isoforms present in the contractile apparatus of muscle will be referred to as muscle tropomyosin, whilst cytoskeletal tropomyosin will be used to describe the isoforms present in the cytoskeleton of all cells.

**Fig. 1:**
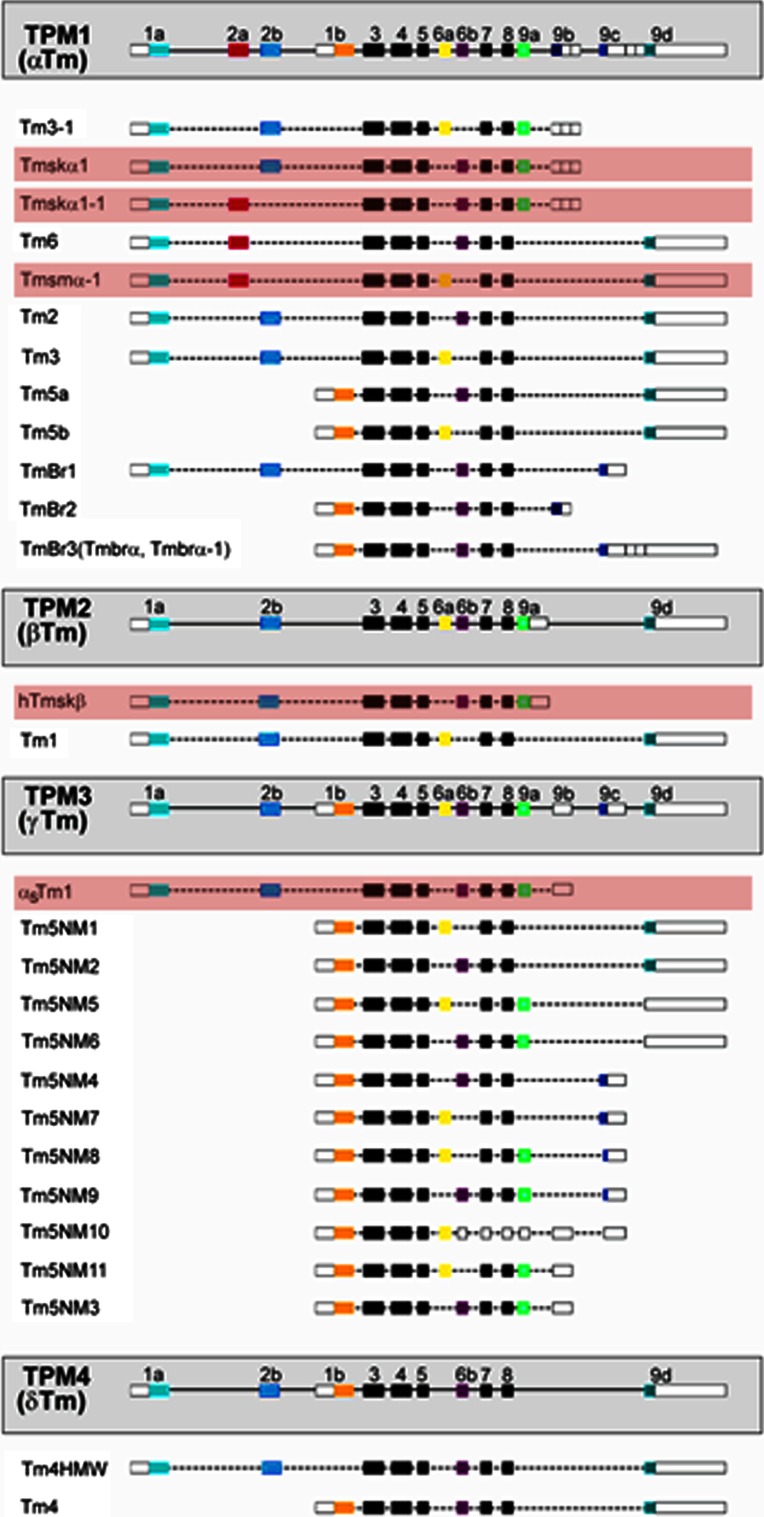
Diagram of the TPM1 (α), TPM2 (β), TPM3 (γ) and TPM4 (δ) genes and the isoforms they encode. The *white boxes* show untranslated regions, *dotted lines* represent introns and the *black boxes* show exons common to all isoforms. Muscle isoforms (shown highlighted in *red*) account for only five tropomyosin isoforms expressed in mammalian cells. Only the major isoforms are included, a number of mRNAs have been detected only by RT-PCR and are not shown

In mammalian cells tropomyosin is encoded by four genes, TPM1, 2, 3 and 4, which through use of multiple promoters and alternative splicing of exons lead to the expression of over 40 isoforms (Pittenger et al. 1994; Dufour et al. 1998; Cooley and Bergtrom 2001). These have historically been classified as either high molecular weight (HMW) (~284 amino acids) or low molecular weight (LMW) (~248 amino acids), that correspond to the use of either exons 1a plus 2 or exon 1b respectively to encode their N-termini (Pittenger et al. 1994). The molecular diversity seen in tropomyosin isoforms comes from the substantial differences seen in alternatively spliced exons (Fig. 1) (Schevzov et al. 2011).

The specialized contractile systems of striated and smooth muscle utilize a total of four actin and only five tropomyosin isoforms (Herman 1993; Pittenger et al. 1994). In contrast, there are just two cytoskeletal actin isoforms and over 40 tropomyosin isoforms found in the cytoskeleton of mammalian cells which facilitate the functional diversity seen in the actin filament system of the cytoskeleton (Gunning et al. 2005, 2008). The role of tropomyosin has been extensively reviewed in Gunning et al. (2005, 2008). In this article we integrate the genetic, molecular cell biology and protein chemistry studies to provide a unifying view of how tropomyosin isoforms act as choreographers of the diversity of function of the animal actin cytoskeleton.

We initially cover the experiments which establish the lack of functional redundancy between tropomyosin isoforms. This leads to consideration of the intracellular spatial segregation of tropomyosin isoforms which provide evidence that these isoforms perform spatially and functionally distinct roles in the cell. The intracellular functional specificity of tropomyosin isoforms is examined in a range of cell types which leads to the conclusion that the spatial segregation of tropomyosins has driven the evolution of functional specialisation. Mechanisms of functional specialisation are then covered with respect to isoform specific interactions with actin binding proteins. Finally, it is proposed that for actin filaments containing tropomyosin, it is the actin-tropomyosin co-polymer which should be considered as the unit of function.

## Tropomyosins are not functionally redundant

### Tropomyosin is essential in yeast

The first work highlighting the essential nature of cytoskeletal tropomyosins was performed in *Schizosaccharomyces pombe* (Balasubramanian et al. 1992). They found that haploid spores carrying the disrupted allele were still able to germinate, though they die soon after as elongated single cells (Balasubramanian et al. 1992). This study also highlighted the essential role that the Cdc8 product, the only tropomyosin isoform present in *S. Pombe*, has in generating the contractile ring required for cytokinesis.

In *Saccharomyces cerevisiae* two tropomyosin isoforms are present; Tpm1p and Tpm2p which are encoded by the TPM1 and TPM2 genes respectively (Drees et al. 1995). Previous work by Liu and Bretscher (1989) had shown that whilst not lethal, the disruption of TPM1 gene expression results in both the disappearance of actin cables and a reduced growth rate. Interestingly whilst a loss of TPM2 gene expression shows no detectable phenotype, disruption of both TPM1 and TPM2 expression results in lethality. This illustrates that in yeast, the expression of at least one isoform is essential for cell viability (Drees et al. 1995). Furthermore it was shown that an elevated expression of the TPM2 gene could not compensate for the loss of TPM1 expression in *S. cerevisiae*, providing evidence for their functional differences (Drees et al. 1995). These results illustrate that in yeast, tropomyosin is essential for cell survival and in the case of *S. cerevisiae*, one isoform performs a specific function that overexpression of the other cannot compensate for.

### Mammalian tropomyosin genes are not redundant and perform essential functions

#### TPM1 (α-) and TPM2 (β-tropomyosin) genes

Homozygous knockout of the TPM1 gene in mice results in embryonic lethality between embryonic day 8.5 and 11.5 (Rethinasamy et al. 1998). Furthermore, it was shown using heterozygous knockout mice that despite a 50 % decrease in mRNA from striated muscle α-Tm in the heart there was no difference in total α-Tm or compensation from other isoforms demonstrated by unaltered levels of the β-Tm protein between heterozygous and control littermates (Rethinasamy et al. 1998). A separate study found that knockout of only the α-Tm striated muscle isoform also results in embryonic lethality, however this occurred between embryonic day 9.5 and 13.5 (Blanchard et al. 1997). These results when taken together suggest that the α-Tm striated muscle isoform and one or more other isoforms encoded for by the TPM1 gene are critical for at least two essential processes in embryonic development. Further work by Wieczorek’s laboratory using transgenic mice demonstrated that changes in relative levels of skeletal tropomyosin in the heart by exchanging striated muscle β-Tm with striated muscle α-Tm does not change the total tropomyosin expression, however the ectopic expression of β-Tm causes severe cardiac pathological abnormalities (Muthuchamy et al. 1995, 1998; Palmiter et al. 1996). This suggests that different tropomyosin isoforms can confer different structural/functional information onto the actin filaments they bind to, allowing them to perform specific functions, and in the heart muscle only striated muscle α-Tm is able to provide the structural/functional information essential for normal cardiac function. It has also been observed that homozygous knockout of the TPM2 gene results in a failure in early developmental processes (Jagatheesan et al. 2010).

#### TPM3 (γ-tropomyosin) gene

Knockout of the TPM3 gene which encodes for 11 cytoskeletal isoforms (Tm5NM1-11) has also been shown to be embryonically lethal in mice by embryonic day 2.5 indicating that at least one LMW product from this gene is essential very early in embryonic development (Hook et al. 2004). This data taken with that from the TPM1 and TPM2 genes illustrates that these genes are not functionally redundant and each is essential for survival. This study also demonstrated that at least the TPM3 gene is essential for embryonic stem cell viability. Given that all four tropomyosin genes are expressed in both embryos and embryonic stem cells, this data demonstrates that the loss in viability is due to the essential functions fulfilled by isoforms from this gene. Further work on the TPM3 gene demonstrated that whilst the deletion of exon 9d-containing isoforms Tm5NM1 and Tm5NM2 lead to partial embryonic lethality in mice, the deletion of the exon 9c-containing isoforms Tm5NM4 and Tm5NM7 does not affect embryonic development. This shows that exon 9d-containing isoforms cannot fully be compensated for by other tropomyosin isoforms in embryonic development (Hook et al. 2011). Whilst the absence of lethality in the exon 9c knockout mice may indicate there is some intragenetic functional redundancy in embryonic development, this does not preclude these isoforms from fulfilling essential functions in later life, or perhaps their function in development may be to help regulate actin filament function in the event of a failure to express other isoforms from the TPM3 gene. Interestingly, deletion of Tm5NM1/2 in stem cells yields no viable stem cells indicating that this subset of isoforms performs at least one essential function (Hook et al. 2011).

### Conclusion

This data provides strong evidence for the idea that multiple tropomyosin isoforms are critical for cell survival. The work performed by Weiczorek’s laboratory using striated muscle β-Tm transgenic mice further demonstrates that the loss or exchange of one isoform for another leads to different functional properties (Palmiter et al. 1996). The lack of redundancy seen between the mammalian genes suggests that these isoforms have different functional properties and that these are required for essential processes in both embryonic development and the maintenance of cellular processes in later life. As a whole these studies support the notion that the regulation of distinct populations of actin filaments by specific tropomyosin isoforms provides a mechanism to fulfill the wide range of specific cellular functions required of the actin cytoskeleton.

## Cytoskeletal tropomyosin isoforms are spatially segregated

### Introduction

The first reports which suggested that tropomyosin isoforms are present at different intracellular locations were by Burgoyne and Norman (Burgoyne and Norman 1985a, b). They first observed that in adrenal chromaffin cells, which express three different tropomyosin isoforms, only one of these was seen to associate with chromaffin granule membranes, suggesting that this specific isoform may be involved in vesicle transport or tethering (Burgoyne and Norman 1985b). They also observed that in neurons, tropomyosins were enriched in cell bodies and dendrites compared to the axons. Thus the nature and composition of the cytoskeletal structures present in the axon and dendrites may differ (Burgoyne and Norman 1985a) and this has been subsequently confirmed in multiple studies (for review, see Gunning et al. 1998a).

Lin et al. (1988) were the first to directly visualize the spatial segregation of isoforms. They reported that whilst both HMW and LMW tropomyosins were seen in stress fibers, only the LMW isoforms were present in ruffling membranes (Lin et al. 1988). These studies have been repeated in a number of cell types and with the ability to see increasing detail and differentiate between more isoforms there has been an increased realization of the extent to which these isoforms are spatially segregated.

### Experimental approaches to isoform sorting

Five independent approaches to isoform sorting have been used by multiple groups to address the question of cytoskeletal-tropomyosin isoform sorting. While there are potential weaknesses with each approach in isolation, the concordance of the multiple approaches has provided confidence that the intracellular sorting of tropomyosin isoforms is an absolute intrinsic property of tropomyosins in all cellular systems in which this has been studied.

Antibodies have been the most widely used tools to approach the sorting of tropomyosin isoforms. The most widely used antibodies have been generated by the Lin and Gunning groups using two completely different strategies. Recent evaluation of the specificity of all the available antibodies from these two groups using panels of purified tropomyosin isoforms has demonstrated that they show a remarkable level of specificity and has also highlighted where care must be taken with potential cross-reactivity (Schevzov et al. 2011). There are now multiple antibodies (polyclonal and monoclonal) available for most isoforms which are used to provide confirmation of results. The biggest concern with antibody studies is the potential of epitope masking due to local conformation changes or binding of associated proteins which obscure the epitope. While antigen retrieval can address this in some situations, it cannot provide absolute certainty that an isoform is absent.

The use of tagged-tropomyosins, usually GFP (or related colours)-derivatives has been widely used to locate and follow individual isoforms. This is most powerful when used in conjunction with antibody staining such as the very careful work of both Temm-Grove et al. (1998) and Tojkander et al. (2011). The combination of both approaches provides very compelling evidence for the specificity of isoform sorting.

Similarly, the use of in situ hybridization to localize specific tropomyosin isoform mRNAs has provided compelling evidence for the intracellular sorting of tropomyosins. Hannan et al. (1995, 1998) demonstrated isoform specific localization of tropomyosin isoform mRNAs which was related to the localization of the corresponding proteins revealed by isoform-specific antibodies. While there was not a one-to-one correspondence of mRNA and protein in neurons in vivo and in vitro, there was a clear concordance of mRNA and protein polarity.

Biochemical sub-fractionation has been used in some cases to detect specific tropomyosins associated with specific intracellular structures/compartments. This was originally used by Burgoyne and Norman (1985b) to demonstrate the association of specific tropomyosins with cromaffin granules and has also been used to confirm the presence of Tm5NM1/2 with Golgi-derived structures (Heimann et al. 1999). The use of sub-fractionation also brings with it potential problems of contamination but is powerful when combined with the other approaches.

Finally, gene knockout or siRNA knockdown experiments have been used to show that removal of the isoform removes antibody staining and/or impacts the function of the compartment containing the isoform. Loss of function can be problematic because of rescue by another isoform but where loss of function is seen, it is most compelling.

Below we consider the wealth of experimental systems and approaches which have unambiguously established the generality of tropomyosin isoform intracellular sorting. The studies documented below have used a range of different approaches or have been confirmed in multiple labs; often using different antibodies or different approaches.

### Differential sorting of Cdc8p in yeast

**Fig. 2:**
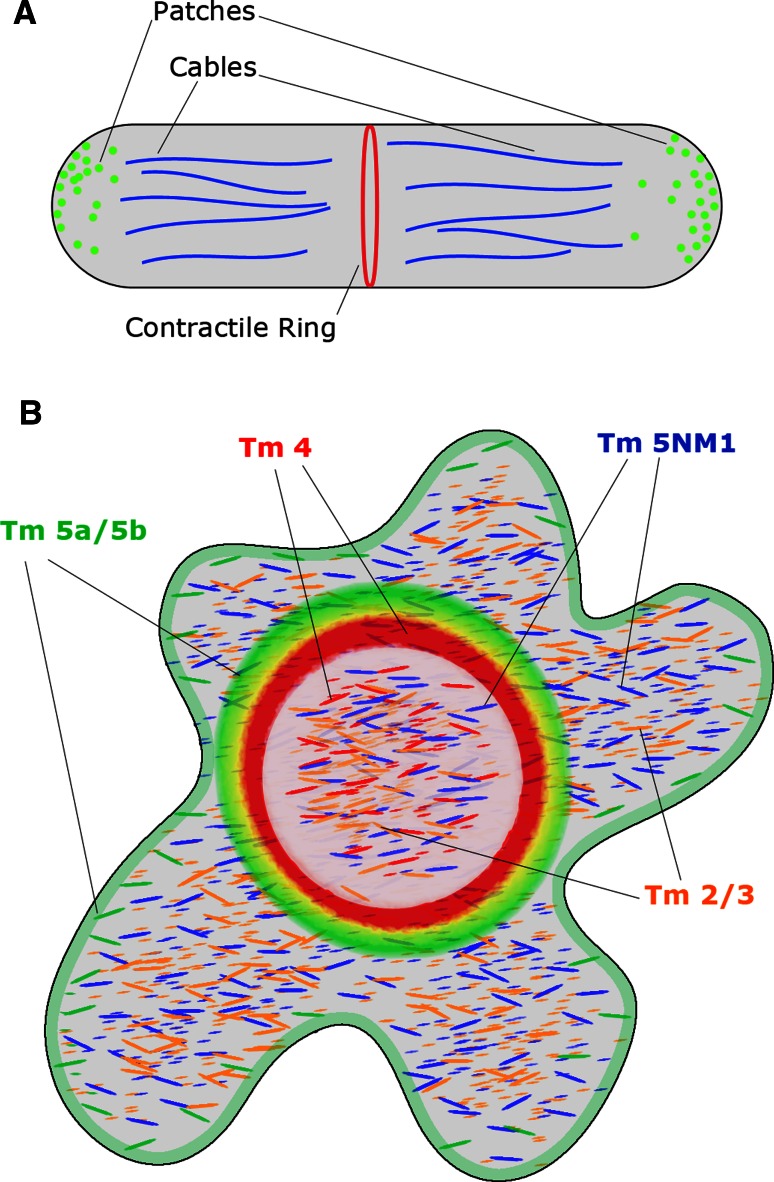
**a** Distribution of tropomyosin in *Schizosaccharomyces pombe*. Actin patches are found near the cell periphery and are not associated with tropomyosin isoforms. The cables which run throughout the cell are associated with unacetylated Cdc8p and favour the binding of myosin-V. In contrast, the contractile ring actin is associated with acetylated Cdc8p which favours the binding of myosin-II. **b** Distribution of tropomyosin in Osteoclasts plated on ivory. Tm4 (*red*) is associated with podosomes (represented as the *inner ring*) and the interior of the cell. Tm5a/5b (*green*) is associated with the F-actin ring (represented as the *outer ring*) and is slightly enriched near the plasma membrane. Whilst some colocalization is observed between Tm5a/5b and Tm4 (*yellow*), Tm5a/5b are notably absent from the podosomes. Both Tm2/3 (*orange*) and Tm5NM1 (*blue*) are both found throughout the cell in different subcellular pools, however the nature of these regions is not yet known

In fission yeast, the acetylation of the only tropomyosin isoform expressed; Cdc8p has a significant impact on its ability to bind and regulate actin filaments (Skoumpla et al. 2007). Coulton et al. (2010) found that acetylated Cdc8p was strongly associated with actin filament bundles in the cytokinetic actomyosin ring (CAR). In contrast, unacetylated Cdc8p was never seen within the CAR and was only associated with filament bundles that extend throughout the cell (Fig. 2a). Since tropomyosins have not been found in either plants or amoebae (Pruyne 2008) these observations demonstrate that sorting tropomyosin isoforms is an intrinsic property that is as old as tropomyosin itself.

### Neurons

Had et al. (1994) compared the localization of two isoforms Tm4 and TmBr3 both in cultured neurons and in the mouse. They found that Tm4 was concentrated at the growth cones of neurons whereas TmBr3 was notably absent from these regions. In vivo, Tm4 was restricted to postsynaptic regions whilst TmBr3 was concentrated at presynaptic sites suggesting that these isoforms fulfill different functional roles in neurons (Had et al. 1994). This spatial segregation has also been detected during neuronal development. In developing neurons Tm5NM1/2 is restricted to the developing axons. However in mature neurons its localization is somatodendritic, and its loss from the axon occurs coincident with the initial appearance of TmBr3 in axons (Weinberger et al. 1996). This temporal regulation has also been seen with Tm5a/5b where its presence in the growth cones of primary neurons diminishes with time in culture (Schevzov et al. 1997).

Further experiments revealed that depolymerization of actin filaments through the addition of cytochalasin B resulted in a loss of spatial segregation of Tm5NM1/2. After wash-out of the drug the spatial segregation of tropomyosin isoforms was restored and Tm5NM1/2 was again mostly absent from the growth cone (Schevzov et al. 1997). This clearly demonstrates that the tropomyosin isoform composition of actin filaments is dependent on the dynamic remodeling of the cytoskeleton.

### Osteoclasts

Osteoclasts possess a highly dynamic cytoskeleton capable of forming a number of distinct intracellular structures which can be defined by the localization of specific tropomyosin isoforms. At the podosomal attachment structures, both Tm4 and Tm5a/5b are present although their localization on these structures is mutually exclusive (McMichael et al. 2006). Whilst Tm4 is found to associate with the interior ends of podosomal actin cores and the top half of F-actin rings in these cells, Tm5a/5b was enriched at the base of podosomal cores and the outer edge of the F-actin rings (McMichael et al. 2006). In contrast, staining for Tm5NM1 and Tm2/3 showed that these isoforms are excluded from attachment structures. Despite both these isoforms being enriched in the cell interior, there was little to no overlap between them (Fig. 2b). It was concluded that they are localized to distinct internal structures in these cells (McMichael et al. 2006). These results indicate that in osteoclasts there are at least four cytoskeletal structures which are associated with specific tropomyosin isoforms.

### Skeletal muscles

Muscle fibers contain three skeletal muscle tropomyosin isoforms which form part of the thin filament where they are involved in the regulation of muscle contraction (Huxley 1973). In addition to skeletal tropomyosin, two cytoskeletal isoforms are expressed which sort to specific compartments within the myofibril. Within the myofibril, Tm5NM1 is specifically sorted to both a filament network adjacent to the Z-line and a subsarcolemma filament system found around the periphery of the myofibril (Kee et al. 2004). Tm4 is also expressed in muscle fibers where it is sorted to two specific locations. Like Tm5NM1, this isoform is sorted to a filament network adjacent to the Z-line where these two isoforms define distinct actin filament populations (Vlahovich et al. 2009). Tm4 is also localized to longitudinal filaments running perpendicular to the Z-line which are associated with muscle fibers undergoing remodeling and repair (Vlahovich et al. 2008).

### Smooth muscle cells

More recently it was found that at least five tropomyosin isoforms are expressed in vascular smooth muscle cells. In addition to the smooth muscle tropomyosin isoforms Tm1 and Tm6 three cytoskeletal isoforms Tm2, Tm5NM1 and Tm4 were also present (Gallant et al. 2011). In contrast to previous work on chicken gizzard smooth muscle by Sanders et al. (1986) in which heterodimer formation was observed, not only did Tm1 and Tm6 not form heterodimers in this cell type but these isoforms also sorted to different intracellular regions and were associated with different actin isoforms (Gallant et al. 2011).

### Fibroblasts

Work in NIH 3T3 fibroblasts also demonstrated that specific tropomyosin isoforms are differentially sorted to specific subcellular locations (Percival et al. 2000). One hour after replating, products from the TPM1 gene were incorporated into stress fiber structures, whereas those from the TPM3 gene were localized to the perinuclear region. This sorting becomes less distinct as the cells progress through the cell cycle. After 8 h isoforms from both genes were localized in stress fibers, however TPM3 isoforms were still present in the central cytoplasm and TPM1 isoforms were more enriched at the cell periphery (Percival et al. 2000). It was later shown that Tm5NM2 specifically sorted to short actin filaments associated with the Golgi complex (Percival et al. 2004).

In primary mouse embryo fibroblasts similar spatial segregation is also seen. Schevzov and colleagues found that the HMW isoforms from the TPM1 gene sorted predominately to stress fibers, whilst Tm5a/5b were the only isoforms specifically located to the ruffling membranes (Schevzov et al. 2005b, 2011).

### Tropomyosin isoforms sort within stress fibers

More recently it has been shown that individual tropomyosin isoforms are further segregated into specific regions along stress fibers. Tojkander et al. (2011) found that only Tm2 was localized along entire stress fibers whilst Tm1, Tm5NM1 and Tm5NM2 were concentrated at the distal ends of filament bundles corresponding to focal adhesions. Tm3 and Tm4 were found proximally to focal adhesions, where they were seen either as short segments or as a dotted pattern. Further results using live-cell imaging demonstrate that tropomyosin isoforms are sequentially recruited to both focal adhesions and dorsal stress fibers and Tm4’s localization to dorsal stress fibers coincides with the incorporation of myosin II into these structures (Tojkander et al. 2011). Furthermore, it was shown that at least four different tropomyosins are required for stress fiber formation (Tojkander et al. 2011).

### Mechanism of isoform sorting

The mechanism of isoform sorting has been the subject of extensive reviews (Gunning et al. 1998a, b, 2005, 2008; Martin and Gunning 2008) which can be summarized very simply. The isoforms are locally assembled and held in place by higher order structures (Martin and Gunning 2008). The site of protein synthesis of isoforms may aid in sorting but does not absolutely determine isoform location (Hannan et al. 1995, 1998). There is no evidence for transport of isoforms to specific intracellular locations (Martin et al. 2010). Hence, the mechanism of sorting most likely occurs at the level of local assembly of the actin filament by mechanism(s) as yet unknown.

### Conclusion

It has been known for years that tropomyosin isoforms are spatially segregated to distinct actin filament populations and this extensive accumulation of data has established that tropomyosin isoform sorting is a fundamental cellular process which is shared across all animal cells.

## Cytoskeletal tropomyosin function

The finely regulated spatial segregation of tropomyosin isoforms is necessary as it ensures that individual isoforms are in the correct locations to fulfill specific functions critical for the normal functioning of the cell.

### Neuronal morphogenesis

Given the fine spatial regulation of tropomyosin isoforms seen in neurons, these cells have been more extensively studied to determine the effects of tropomyosin on neuronal morphogenesis. Primary neurons from transgenic mice overexpressing Tm5NM1 were found to have increased neuronal branching in both dendrites and axons and a significant increase in growth cone size without any noticeable change in its gross morphology (Schevzov et al. 2005a). In contrast, neurons from transgenic mice overexpressing Tm3 had both significantly decreased numbers and length of dendrites. These results indicate that Tm5NM1 and Tm3 contain different structural information, and their expression gives rise to filament populations with different functional properties (Schevzov et al. 2005a). Consistent with the increase in dendritic length seen with Tm5NM1 overexpression, neurons from mice lacking Tm5NM1/2 were seen to have a significant decrease in dendritic length, as well as a number of other morphological changes compared to control neurons (Fath et al. 2010). These results show that altering the tropomyosin composition of filaments in neurons leads to significant changes in neuronal morphogenesis.

### Trafficking

Organelle transport plays an essential part in many cellular functions and is a process which relies on both actin filaments and microtubules. Pelham et al. (1996) investigated the role of tropomyosin in organelle transport through the microinjection of Tm3 into NRK cells. They found that the microinjection of Tm3 but not Tm5NM1 causes a remarkable redistribution of membrane-bound organelles into the perinuclear region (Pelham et al. 1996). These results at the very least indicate that these two isoforms are functionally distinct.

### CFTR membrane levels

The actin cytoskeleton has also been shown to play a role in the delivery of the cystic fibrosis transmembrane conductance regulator (CFTR) into the apical membrane of epithelial cells. It has been shown that reduced expression of Tm5a and Tm5b result in an increased surface expression of CFTR in vitro indicating that these isoforms may be associated with a subpopulation of actin filaments directly involved in the removal of CFTR from the plasma membrane (Dalby-Payne et al. 2003). It was concluded that these isoforms play a role in the regulation of endocytosis.

### Cytokinesis

A number of studies have shown that tropomyosin plays an important part in cytokinesis. In yeast the role that tropomyosin plays in the regulation of cytokinesis has been extensively studied. Balasubramanian et al. (1992) found that the tropomyosin isoform Cdc8 was essential for cell survival in *S. pombe*. Whilst Cdc8 was not required for spore germination, cell growth or DNA replication it is essential for cytokinesis. This indicates that the essential role for this protein is to form part of the F-actin contractile ring (Balasubramanian et al. 1992). Further work by Mulvihill’s laboratory showed that the function and sorting of Cdc8 to different cellular structures was dependent upon its acetylation (Skoumpla et al. 2007; Coulton et al. 2010). The cytokinetic deficit found in cells which lack the NatB *N*-α-acetyltransferase regulatory subunit was also shown to be as a result of a lack of tropomyosin acetylation (Coulton et al. 2010). Stark et al. (2010) found that this regulation of cytokinesis in *S. pombe* was through its role in stabilizing actomyosin interactions.

The regulation of cytokinesis by tropomyosin has also been seen in mammalian cells. Hughes et al. (2003) examined tropomyosin expression in developing and neoplastic brain tissue. They found that in the embryonic brain HMW tropomyosin expression was restricted to proliferative areas, whereas in the adult brain, staining could only be seen in blood vessels. They also noted that in rare proliferating astrocytes HMW tropomyosins were found in the contractile ring, but after withdrawal from the cell cycle HMW tropomyosin expression was down regulated (Hughes et al. 2003). Forced expression of Tm5NM1 and a chimeric tropomyosin Tm5/3 in Chinese hamster ovary cells resulted in faster cell division which would suggest that tropomyosin is necessary for the formation of the contractile ring (Eppinga et al. 2006). The abnormal division seen in Tm5NM1 overexpressing cells may be due to the inability of other actin binding proteins to disassemble the contractile ring. This is supported by the observation that Tm5NM1 excludes the association of ADF with filaments containing this tropomyosin isoform (Bryce et al. 2003).

### Podosomes in osteoclasts

Osteoclasts express several cytoskeletal isoforms which sort to specific regions. Tm4 was found to be associated with the core of podosomes, suggesting that it may play a role in regulating these structures (McMichael et al. 2006). Knockdown and overexpression studies revealed a direct role for Tm4 in regulating both podosomal and sealing zone actin filaments. McMichael and Lee (2008) found that either under- or overexpression of Tm4 disrupted these attachment structures leading to impaired bone resorption and cell motility. Further work examining the role of Tm2 and Tm3 in these cells revealed that despite a lack of association with distinct actin structures in these cells, these isoforms play a role in the regulation of osteoclast morphology and function (Kotadiya et al. 2008). These results taken together demonstrate that individual isoforms decorate distinct actin filament populations in osteoclasts, giving them specific functional properties necessary for normal cellular function.

### Stem cell viability

A number of tropomyosin isoforms play a critical role in the regulation of stem cell viability and embryonic development. Eliminating the cytoskeletal isoforms from the TPM3 gene (Tm5NM1-11) results in lethality prior to embryonic day 2.5 and the inability to generate viable stem cells (Hook et al. 2004). Embryonic stem cells deleted for exons 9a/9b of the TPM3 gene (Tm5NM3, 5, 6, 8, 9, 11) are viable whereas the failure to generate viable stem cells lacking exon 9d of the TPM3 gene (Tm5NM1,2) indicates that at least one of these isoforms is essential for cell growth in vitro (Hook et al. 2011). Their role in cell growth is further supported by the fact that these isoforms are expressed in most, if not all cells and there is an increased reliance on these isoforms in almost all forms of cancer (Stehn et al. 2006). These results demonstrate that isoforms from the TPM3 gene are required for the normal functioning of a cell and cannot be compensated for by products from the other three genes.

### Excitation contraction coupling in skeletal muscle fibers

The importance of cytoskeletal tropomyosin isoforms in cellular function is seen in vivo in skeletal muscle fibers. In muscles from mice null for Tm5NM1 the level of T-tubule dysmorphology was increased when compared to WT muscles, and the Tm5NM1 KO mouse muscles had altered contractile properties which were not due to fiber-type changes (Vlahovich et al. 2009). Further experiments revealed the altered contractile performance was a result of dysregulation of T-tubule function due to the loss of Tm5NM1. This demonstrates that the LMW Tm4 expressed in an adjacent location in muscle cannot compensate for the loss of Tm5NM1.

### Conclusion

The specialized function of tropomyosin isoforms described here provides clear evidence that tropomyosin has a much underappreciated role in the functional regulation of the actin cytoskeleton. In light of this, it would seem highly likely that the binding of individual tropomyosin isoforms to actin filaments confers specific functional properties upon these filaments. This gives rise to distinct filament populations localized to specific regions of a cell where they perform different functions.

## Mechanisms of specialized tropomyosin function

Historically, tropomyosin has been both studied and understood in terms of its ability to regulate the myosin II interaction with the actin filament in muscle (Murray and Weber 1973). In vitro studies revealed that chicken gizzard tropomyosin displays a greater cooperativity than rabbit skeletal tropomyosin in terms of their effects on myosin subfragment 1 activity (Lehrer and Morris 1984). Additional protein chemistry studies also revealed that muscle tropomyosin could regulate muscle actin filament stability (Fujime and Ishiwata 1971) and inhibit both DNase 1 (Hitchcock et al. 1976) and cofilin (Bernstein and Bamburg 1982) induced depolymerisation of muscle actin. The implications of this work for the functional diversity of the cytoskeleton required the development of molecular genetic approaches to manipulate the composition of the cytoskeleton and visualization of different filament populations.

### Evolutionary consequences of isoform sorting

The ability to sort isoforms to different spatial, and therefore functional contexts, will inevitably result in the divergence of their functional capacities due to the differing functional constraints placed upon them. This sorting will lead to the specialized functions of intracellular sites regulated by the specific isoform population present. There is a clear lack of functional redundancy seen between different tropomyosin isoforms, and they show finely tuned spatial segregation to specific subcellular locations (Martin and Gunning 2008). This suggests that the creation of different tropomyosin isoforms throughout evolution allows for the creation of a range of actin filament populations which possess the structural information required to fulfill a broad range of unique functions (Gunning et al. 2008).

### Regulate actin polymer levels

A number of studies have demonstrated the ability of tropomyosin to regulate levels of F-actin within cells. Schevzov et al. (2008) found that following the overexpression of Tm3 and Tm5NM1, the levels of other cytoskeletal tropomyosin isoforms and β- and γ-actin levels were unchanged in transgenic tissues. Interestingly in primary hippocampal neurons from Tm5NM1 transgenic mice, enrichment of Tm5NM1 staining in the growth cones was associated with a significant increase in both total phalloidin signal and mean pixel intensity (Schevzov et al. 2008). Similar results were reported in osteoclasts where manipulation of Tm4 expression resulted in changes in F-actin levels at the site of Tm4 localization. Overexpression of Tm4 caused an increase in F-actin in podosomes, whereas the knockdown of this isoform resulted in significant thinning of F-actin in the actin ring and sealing zone (McMichael and Lee 2008). These findings indicate that the levels of cytoskeletal tropomyosin are limiting for actin polymerization in the subcellular regions where these isoforms are sorted. Thus, tropomyosin isoform sorting regulates total actin polymer levels at specific intracellular locations.

### Regulate myosin motors

Whilst the regulation of myosin driven contraction by tropomyosin has been extensively studied in striated muscle, comparatively little is known about the interactions between tropomyosin dependent regulation of actomyosin interactions in cytoskeletal systems. Work by Fanning et al. (1994) illustrated that the ability of tropomyosin to regulate the ATPase activity and translocation of muscle myosin II along actin filaments was dependent on the isoform present. In contrast, all tropomyosin isoforms tested inhibited the ATPase activity and translocation of myosin I to a similar extent. This demonstrated that regulation of myosin motor interactions with actin was isoform dependent and that a single isoform can have opposing effects on different myosin motors.

In fission yeast the tropomyosin isoform Cdc8p was found to enhance myosin II motor activity, promoting the formation of the contractile ring (Stark et al. 2010). Further work by Lord’s laboratory has shown that Cdc8p also regulates the activity of myosin V (Clayton et al. 2010). Coulton et al. (2010) found that whilst acetylation of Cdc8p was required for the regulation of myosin II, there was no effect on the regulation of myosin I or V. Recent work has demonstrated that tropomyosin allows for the processive movement of class V myosins in *S. cerevisiae*. Hodges et al. (2012) found whilst class V myosin motors are unable to move processively along bare skeletal muscle actin, supporting previous in vitro studies, the creation of a more biologically relevant filament through the addition of tropomyosin allowed for the processive movement of Myo2p. This data strongly supports the idea that actomyosin interactions are sensitive to the presence of tropomyosin along the filament.

The regulation of myosin has also been seen in more complex mammalian cells. Bryce et al. (2003) found that myosin IIA, but not IIB, was recruited to stress fibers in Tm5NM1 overexpressing B35 cells resulting in a substantial increase in myosin II activity. The recruitment of myosin IIA was also seen in the dendrites of cortical neurons from transgenic mice overexpressing Tm5NM1 (Bryce et al. 2003). However, in growth cones where myosin IIA is absent, IIB was able to associate with Tm5NM1 containing filament bundles demonstrating that the preferential recruitment of myosin is dependent upon the availability of specific myosin isoforms (Schevzov et al. 2005a). Tang and Ostap (2001) provided further evidence for the regulation of myosin I by tropomyosin. They found that the exclusion of myosin I from actin structures that contain tropomyosin was due to the regulation of the actomyosin interaction by tropomyosin.

### Regulate interactions of other actin binding proteins

#### Actin depolymerizing factor/cofilin

Actin depolymerizing factor (ADF)/cofilin depolymerizes actin filaments and was initially found to compete with tropoymosin for binding to the filament (Bernstein and Bamburg 1982). Bryce et al. (2003) found that this antagonistic interaction was isoform specific. Tm5NM1 expressing cells had an increased level of phosphorylated ADF indicating that it was displaced from actin filaments, however TmBr3 recruits ADF to the lamellapodia where they colocalize on the same filaments. Thus, tropomyosins can be seen as collaborators or competitors of ADF/cofilin depending on the tropomyosin isoform (Kuhn and Bamburg 2008).

#### Fascin

Fascin is an actin bundling protein found in stress fibers and filipodia which localizes with HMW but not LMW tropomyosin isoforms (Yamashiro-Matsumura and Matsumura 1986). In control B35 neuroblastoma cells there is an association of fascin with Tm2. Creed et al. (2011) found that in Tm3 overexpressing cells there was a significant shift in fascin association from Tm2 to Tm3-containing filaments. Furthermore they found that the overexpression of Tm3 resulted in an increase in fascin expression. This indicates that different tropomyosin isoforms can alter the expression of endogenous actin binding proteins, possibly via changing the partitioning of these proteins between the soluble and filament bound pools and hence their turnover kinetics.

#### Formin homology proteins

Formins are a diverse family of actin nucleating proteins. Whilst there have been several biochemical studies which investigate the kinetics of the interaction between formins and tropomyosin (Wawro et al. 2007; Ujfalusi et al. 2009, 2012), there has been little work describing this interaction in a cell based system. In fission yeast, the formin Cdc12p nucleates actin filaments which the tropomyosin isoform Cdc8p binds to with diverse effects on Cdc12p-mediated actin assembly (Skau et al. 2009). Cdc8p’s binding both increases the rate of elongation and allows annealing of the filaments before stopping Cdc12p-mediated elongation. Interestingly, Cdc8p may then stop Cdc12p-mediated elongation through either the trapping of Cdc12p or dissociating it from the filament (Skau et al. 2009). This intricate relationship is still not fully understood and it is likely that the interactions which occur in mammalian cells, which express multiple formin and tropomyosin isoforms, would be far more complex. Whilst the consequences of this are not known, this study suggests that tropomyosin’s role in the regulation of the cytoskeleton is far more complex and intricate than previously thought.

### Responsive to availability of active ABPs

More recently it was shown that the HMW isoform Tm3 independently regulates the function of actin filaments at specific intracellular sites (Creed et al. 2011). They found that active ADF/cofilin was localized in the cell body and within the base of the filipodia in Tm3 overexpressing cells, whilst the inactive phosphorylated ADF/cofilin was only found in the perinuclear region. This indicates that only the active ADF/cofilin is able to be recruited to Tm3-containing filaments. Furthermore they demonstrated that inactivation or knockdown of ADF/cofilin caused a change in cell morphology and cytoskeletal organization more resembling the control B35 cells (Creed et al. 2011). This indicates that whilst different tropomyosin isoforms will preferentially recruit specific ABPs, their impact on the cell is also determined by the local availability of active ABP’s.

## The actin-tropomyosin copolymer as the unit of function

Early research into understanding the cytoskeleton was dominated by protein biochemistry and in vitro studies, which have been critical in providing a basis for in vivo studies. However, whilst in vitro work has been fundamental in establishing many of the key concepts known today and has driven the model building surrounding interactions of cytoskeletal proteins, there have been numerous cases where the biochemical data has not been supported by molecular genetics. Interpretation of in vitro observations has established a view that filaments are generic and the specific functional outcomes observed within cells are due to the chance interaction of a large number of actin binding proteins with filament bundles at any given time. However, many of these experiments were performed in a fixed environment, under ideal conditions using α-skeletal, not cytoskeletal actin and in the absence of actin binding proteins needed to assemble filaments into the biological structures seen in vivo. In contrast, molecular genetics and in vivo data have driven functional biology. The sorting of actin binding proteins gives rise to filament bundles with unique functional information that is critical for the functioning of a cell.

Whilst many biochemical studies have demonstrated both polymerization of actin and subsequent binding of cytoskeletal actin binding proteins to skeletal actin in vitro, how these processes occur in vivo is yet to be established. There is an ever increasing body of evidence to suggest that there is no active transport for the movement of tropomyosin to specific spatial regions, and that its sorting relies on the active formation of filaments. This suggests a ‘molecular sink’ model whereby isoforms accumulate in structures where they are most stable. This hypothesis has been supported by drug studies demonstrating that a loss of filaments results in the abolishment of isoform sorting. Intrinsic to the sorting of tropomyosin is that tropomyosin binds to polymerizing actin filaments, both providing a mechanism of stabilizing single filaments and facilitating the interactions with surrounding actin binding proteins.

Whilst the notion of gestalt-binding proposed by Holmes and Lehman (2008) would accurately predict the binding of tropomyosin to actin filaments in vitro, it fails to take into account the complexity of the cellular environment. The formation of filaments in a cellular environment is a continuous process that occurs throughout the whole cell. Therefore, the notion that an actin filament forms in an environment free from interactions with the plethora of actin binding proteins present throughout the cytoplasm would seem a highly inefficient process. On the other hand, the binding of specific tropomyosin isoforms to nucleated actin filaments whose conformational twist favors that particular isoform, would allow not only for the stabilization of the growing filament, but for the formation of a functionally distinct filament population already equipped with the structural information required to fulfill a specific functional role. Given the dynamic nature of actin filaments in an intracellular environment (for example, see Tojkander et al. 2011) it seems more likely that specific actin and tropomyosin isoforms co-assemble to form functionally distinct filaments. The mechanism of assembly may be influenced by the nature of filament nucleation in a cell which differs from that used in most in vitro studies.

The proposal that tropomyosin plays a somewhat redundant role in cytoskeletal regulation where its binding can be so easily disrupted by other binding proteins is questioned by the presence of a number of essential tropomyosin isoforms within the cell. The finely regulated sorting of tropomyosin to distinct filament populations, highly regulated both in time and space would indicate that its role is far more important than a ‘parking attendant’ for actin filaments. Instead the overwhelming accumulation of data now proposes a more biologically relevant model for the tropomyosin dependent regulation of the actin cytoskeleton (Gunning et al. 2008).

From the results discussed in this review, it would seem obvious in retrospect, that tropomyosin must play a key role in the regulation of the actin cytoskeleton. The two cytoskeletal actin isoforms; β- and γ-actin vary by only a few amino acids, presumably as further changes to the highly conserved amino acid sequence would result in an inability to provide adequate structural support. In contrast, there are over 40 tropomyosin isoforms that are extensively regulated both spatially and temporally. The differential sorting of these structurally distinct isoforms to specific subcellular locations allows for the formation of functionally distinct actin filament populations, each possessing unique structural information.

**Fig. 3:**
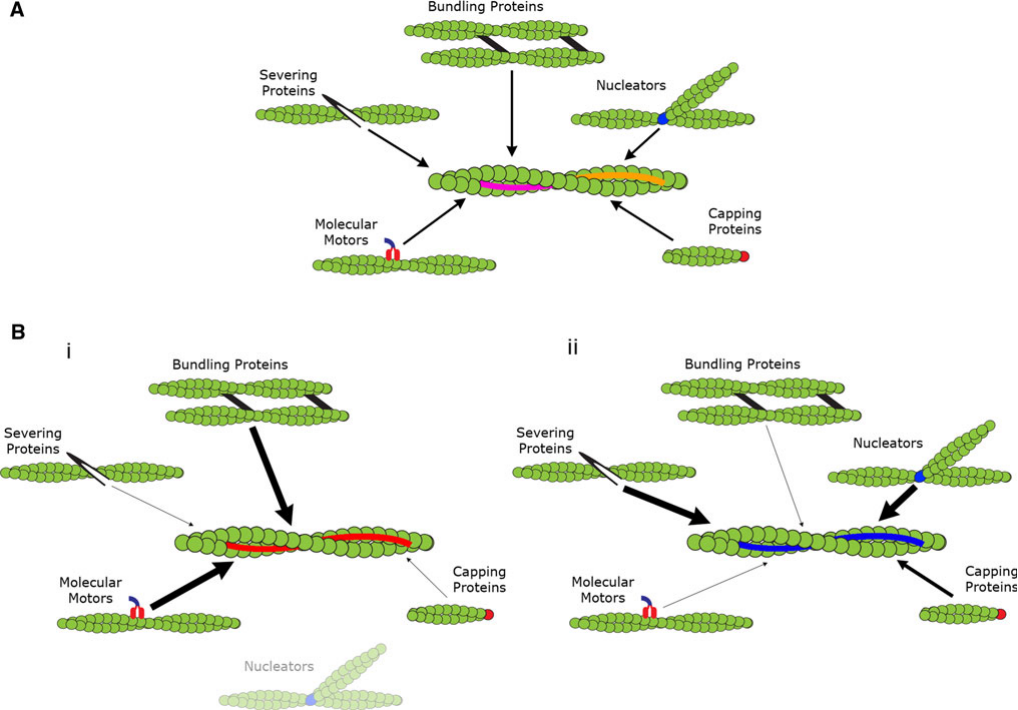
**a** Previous view: Actin filaments are formed independent of any association with actin binding proteins aside from their nucleators. Once formed tropomyosin isoforms nonspecifically dimerize with any isoform of the same molecular weight. At a critical concentration these homo- or heterodimers will form heteropolymers along the filament where they act only to stabilize the actin filament. **b** Current model: Actin filaments are nucleated and begin to polymerize. Specific tropomyosin isoforms co-polymerize with the newly nucleated filaments stabilizing them whilst polymerization continues. Once the mature filament has formed the bound tropomyosin regulates the interaction between actin and actin binding proteins. (*i*) Seen above the *red* decorated filaments there is enhanced binding of bundling proteins and molecular motors, whilst nucleators and capping protein interaction is greatly diminished. There is also complete exclusion of severing proteins from these structures. (*ii*) In contrast, filaments decorated by *blue* tropomyosin enhance the severing protein’s binding whilst modulating the access of other actin binding proteins independent of the *red* filaments. This allows for the formation of multiple filament populations which are able to perform specific cellular functions and that are independently regulated in different regions of the cell

Here the various tropomyosin isoforms act primarily as choreographers regulating the dynamic interaction between actin and all its binding proteins. This dynamic system gives rise to the formation of unique and functionally distinct filament systems largely based on one gene family. From an evolutionary perspective, cells which were able to utilize multiple filament systems independently regulated by single tropomyosin isoforms would be given a distinct advantage. This would likely have driven the evolution of the tropomyosin dependent regulation of the actin cytoskeleton and the basis for the functional diversity that has become characteristic of the actin filament system. This is described graphically in Fig. 3.

## Abbreviations

HMW: High molecular weight
LMW: Low molecular weight
ABPs: Actin binding proteins
